# [^68^Ga]FAPI PET for Imaging and Treatment Monitoring in a Preclinical Model of Pulmonary Fibrosis: Comparison to [^18^F]FDG PET and CT

**DOI:** 10.3390/ph17060726

**Published:** 2024-06-03

**Authors:** Hao Ji, Xiangming Song, Xiaoying Lv, Fuqiang Shao, Yu Long, Yangmeihui Song, Wenyu Song, Pengxin Qiao, Yongkang Gai, Dawei Jiang, Xiaoli Lan

**Affiliations:** 1 Department of Nuclear Medicine, Union Hospital, Tongji Medical College, Huazhong University of Science and Technology, Wuhan 430022, China; hyxjih@126.com (H.J.); sxm960520@163.com (X.S.); 15927506827@163.com (X.L.); m15228213350@163.com (F.S.); longyu96_18@163.com (Y.L.); songymh@hust.edu.cn (Y.S.); wenyusong@hust.edu.cn (W.S.); pxqiao@zzu.edu.cn (P.Q.); gykmail@hust.edu.cn (Y.G.); 2 Hubei Key Laboratory of Molecular Imaging, Wuhan 430022, China; 3 Key Laboratory of Biological Targeted Therapy of the Ministry of Education, Wuhan 430022, China

**Keywords:** fibroblast activating protein inhibitor, idiopathic pulmonary fibrosis, positron emission tomography, diagnosis, treatment monitoring

## Abstract

Purpose: This study aimed to evaluate the feasibility of using [^68^Ga]-fibroblast-activating protein inhibitor (FAPI) positron emission tomography (PET) imaging for diagnosing pulmonary fibrosis in a mouse model. We also examined its value in monitoring treatment response and compared it with traditional [^18^F]-fluorodeoxyglucose (FDG) PET and computed tomography (CT) imaging. Methods: A model of idiopathic pulmonary fibrosis was established using intratracheal injection of bleomycin (BLM, 2 mg/kg) into C57BL/6 male mice. For the treatment of IPF, a daily oral dose of 400 mg/kg/day of pirfenidone was administered from 9 to 28 days after the establishment of the model. Disease progression and treatment efficacy were assessed at different stages of the disease every week for four weeks using CT, [^18^F]FDG PET, and [^68^Ga]FAPI PET (baseline imaging performed at week 0). Mice were sacrificed and lung tissues were harvested for hematoxylin-eosin staining, picrosirius red staining, and immunohistochemical staining for glucose transporter 1 (GLUT1) and FAP. Expression levels of GLUT1 and FAP in pathological sections were quantified. Correlations between imaging parameters and pathological quantitative values were analyzed. Results: CT, [^18^F]FDG PET and [^68^Ga]FAPI PET revealed anatomical and functional changes in the lung that reflected progression of pulmonary fibrosis. In untreated mice with pulmonary fibrosis, lung uptake of [^18^F]FDG peaked on day 14, while [^68^Ga]FAPI uptake and mean lung density peaked on day 21. In mice treated with pirfenidone, mean lung density and lung uptake of both PET tracers decreased. Mean lung density, [^18^F]FDG uptake, and [^68^Ga]FAPI uptake correlated well with quantitative values of picrosirius red staining, GLUT1 expression, and FAP expression, respectively. **Conclusions:** Although traditional CT and [^18^F]FDG PET reflect anatomical and metabolic status in fibrotic lung, [^68^Ga]FAPI PET provides a means of evaluating fibrosis progression and monitoring treatment response.

## 1. Introduction

Idiopathic pulmonary fibrosis (IPF) is a progressive disease characterized by abnormal activation of myofibroblasts, which induces injury of lung epithelium or endothelium via excessive deposition of collagen and extracellular matrix proteins. The resulting interstitial fibrosis eventually leads to dyspnoea, serious pulmonary dysfunction, and even death [1,2,3]. Survival of late-stage IPF patients ranges from 2 to 4 years. The only effective treatment for late-stage disease is lung transplantation; although pirfenidone and nintedanib have been approved for IPF treatment, they can only delay its progress [4].

Early diagnosis and intervention can improve outcomes [5]. However, this can be difficult because the disease is frequently clinically silent in the early stages and no reliable IPF biomarkers have been identified. IPF is currently diagnosed via lung biopsy, which is associated with procedural risks, and high-resolution computed tomography (HRCT) [6]. Usual interstitial pneumonia (UIP) can be diagnosed with HRCT; however, imaging does not provide data regarding molecular alterations and can just show the anatomical changes of organ structure with high spatial resolution, it cannot distinguish active and quiescent lesions [7,8]. For patients with non-typical IPF, lung biopsy is advised [9]. Identification of molecular imaging biomarkers of IPF could preclude the need for biopsy and assist with diagnosis.

Positron emission tomography (PET) can visualize biological processes at the molecular level in a real-time quantitative manner. [^18^F]-fluorodeoxyglucose ([^18^F]FDG) imaging can reveal glucose uptake and metabolism in the lung, which is increased in patients with IPF [10,11,12,13]. The increase in lung density on HRCT is proportional to the increase in lung uptake of [^18^F]FDG [12,14,15]. However, background [^18^F]FDG uptake in the lung can cause false-positive results. In addition, anti-fibrotic drugs such as pirfenidone and nintedanib can affect [^18^F]FDG PET and CT imaging. Treatment selection and monitoring the patient’s response must be individualized.

The use of new probes targeting predominant cells and specific cell products involved in the pathogenesis of pulmonary fibrosis and reflecting the microenvironment change in the pulmonary fibrosis may overcome the deficiencies of [^18^F]FDG PET/CT and CT in disease diagnosis and monitoring antifibrosis efficacy. Collagen binding probes [^68^Ga]Ga-CBP7 [16], [^68^Ga]Ga-CBP8 [17] targeting to collagen, [^177^Lu]Lu-DOTA-RGD targeting to integrin α_v_β_3_ [18], [^18^F]FMISO revealing the hypoxic microenvironment development [19], and [^177^Lu]Lu-DOTA-NOC targeting to somatostatin receptors on inflammatory cells [18], [^99m^Tc]Tc-rhAnnexin V-128 [20], and [^18^F]F-ML-10 [21] targeting to apoptotic cells, and [^18^F]F-AzaFol [22] targeting to folic acid receptors to display macrophages have been investigated.

Fibroblast activation protein (FAP), a non-classical serine protease, is highly expressed in cancer-associated fibroblasts [23] and up-regulated in remodeling tissues in autoimmune and fibrotic diseases [24]. Therefore, fibroblast activation and proliferation might be closely related to the occurrence and development of these diseases. [^68^Ga]-fibroblast activating protein inhibitor ([^68^Ga]FAPI), a PET tracer that specifically targets to FAP, has been widely used for imaging of tumors; fibrotic diseases of the heart [25], liver [26] and kidney [27]; and autoimmune diseases [27,28]. However, limited studies of radionuclide labelled FAPI PET in pulmonary fibrosis have been reported, such as the study of Cong-Ying Song et al. [29].

FAP-α is selectively induced in areas of IPF undergoing tissue remodeling [30]. Moreover, in a mouse model of pulmonary fibrosis, FAP is involved in matrix metalloproteinase-mediated extracellular matrix remodeling [31]. Of note, FAP is expressed almost exclusively in collagen-producing fibroblasts [32]. Therefore, we hypothesized that [^68^Ga]FAPI can be used to monitor fibroblast activation and infiltration in the pulmonary fibrosis setting. This study aimed to evaluate the feasibility of using [^68^Ga]FAPI PET for diagnosing pulmonary fibrosis in a mouse model of pulmonary fibrosis, examine its value in monitoring treatment response, and compare it with traditional [^18^F]FDG PET and CT imaging.

## 2. Results

### 2.1. [^68^Ga]FAPI PET Detects BLM-Induced Lung Fibrosis

**Figure 1:**
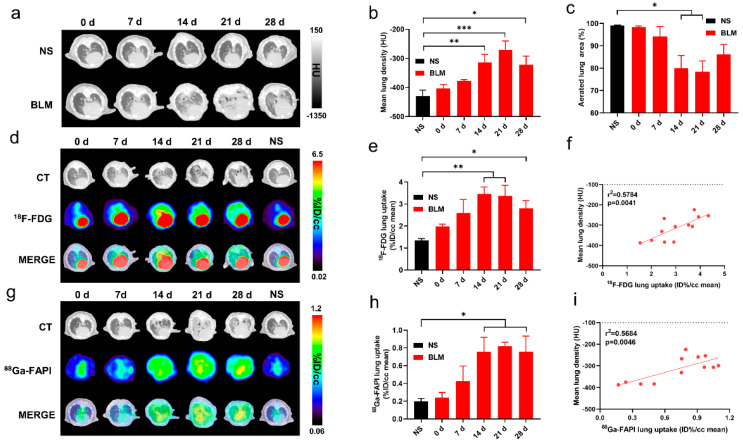
CT, [^18^F]FDG and [^68^Ga]FAPI PET imaging could detect BLM-induced lung fibrosis. (**a**) Representative lung CT images of different mice on day 0, 7, 14, 21, and 28 in the control group and BLM group, respectively. (**b**) Mean lung density quantified on CT images at different time points. (**c**) The percentage of aerated lung area (HU < −100) on CT images. (**d**) Lung CT and [^18^F]FDG and the fusion images ([^18^F]FDG PET/CT) day 0, 7, 14, 21, and 28 in the control group and BLM group, respectively. (**e**) The mean [^18^F]FDG lung uptake (%ID/g) quantified on [^18^F]FDG PET/CT images at different time points. (**f**) Correlation between mean lung density (HU) measured on CT images and [^18^F]FDG lung uptake (%ID/g) of measured on PET. (**g**) Lung CT and [^68^Ga]FAPI and the fusion images at day 0, 7, 14, 21, and 28 in the control group and BLM group, respectively. (**h**) The mean [^68^Ga]FAPI lung uptake (%ID/g) quantified on [^68^Ga]FAPI PET/CT images at different time points. (**i**) Correlation between mean lung density (HU) measured on CT images and [^68^Ga]FAPI lung uptake (%ID/g) measured on PET. NS means control group, and the mean value of the control group was obtained at day 28. The results are presented as mean ± SEM (*n* = 4 for NS and *n* = 3–4 for BLM groups). * *p* < 0.05 and ** *p* < 0.01, *** *p* < 0.001.

CT and PET imaging using either [^18^F]FDG or [^68^Ga]FAPI enabled detection of pulmonary fibrosis. Initially, lung uptake and the pulmonary exudation and consolidation regions in the BLM group increased then decreased slowly (Figure 1a,d,g). Significant differences in lung density and [^18^F]FDG or [^68^Ga]FAPI uptake were found between the control and IPF groups (Figure 1b,e,h). Mean lung density (MLD), non-aerated lung area percentage, and [^68^Ga]FAPI lung uptake peaked on day 21, while [^18^F]FDG lung uptake peaked on day 14. These results confirmed that CT can visualize interstitial infiltrates in the lung and that [^18^F]FDG and [^68^Ga]FAPI PET imaging can detect IPF. Presumably, [^18^F]FDG shows metabolic changes during IPF progression, while [^68^Ga]FAPI reflects changes in fibrosis. Although the uptake of both tracers increased as IPF progressed, the different peak times suggest different pathological processes (Figure 1e,h). [^18^F]FDG and [^68^Ga]FAPI lung uptake correlated well with MLD (Figure 1f,i), and MLD values increased in conjunction with increased area of non-aerated lung (Figure 1c).

### 2.2. [^68^Ga]FAPI PET Correlates with Pathological Findings

**Figure 2:**
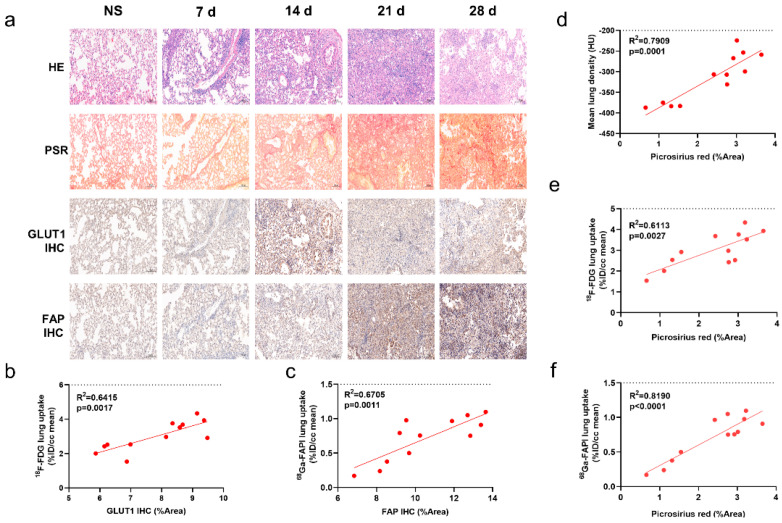
CT, [^18^F]FDG and [^68^Ga]FAPI PET correlated with the pathological findings of pulmonary fibrosis. (**a**) Lung hematoxylin-eosin (H&E) staining, picrosirius red (PSR) staining, immunohistochemical staining (IHC) of glucose transporters 1 (GLUT1), and fibroblast-activation protein (FAP) in the control group (NS) and BLM group at day 0, 7, 14, 21, and 28, and pathological sections of the control group were obtained at day 28. (**b**) Correlation between the lung [^18^F]FDG uptake (%ID/cc) with percentage positive area (%Area) of GLUT1 IHC. (**c**) Correlation between the lung [^18^Ga]FAPI uptake (%ID/cc) with percentage positive area (%Area) of FAP IHC. (**d**–**f**) Correlation between the mean lung density (MLD), [^18^F]FDG and [^68^Ga]FAPI lung uptake (%ID/cc) and picrosirius red staining (%Area). (*n* = 4 for NS and *n* = 3–4 for BLM groups, *p* < 0.05 represents statistically significant).

Degree of inflammatory cell infiltration, alveolar epithelial cell hyperplasia, and alveolar collapse differed between the control and IPF groups (Figure 2a). PSR staining demonstrated a broad distribution of collagen in the lung starting on day 14. GLUT1 was mainly expressed on inflammatory cells and some fibroblasts. Fibroblasts stained positive for FAP.

Similar to the uptake of [^18^F]FDG and [^68^Ga]FAPI, collagen concentration and levels of GLUT1 and FAP expression initially increased then decreased (Supplemental Materials Figure S1a–c). Notably, lung uptake of [^18^F]FDG and [^68^Ga]FAPI correlated well with levels of GLUT1 (Figure 2b) and FAP (Figure 2c) expression, respectively; in addition, [^18^F]FDG and [^68^Ga]FAPI uptake and MLD correlated with PSR staining (Figure 2d–f). These correlations suggest that the imaging findings truly reflect pathological alterations. [^68^Ga]FAPI lung uptake correlated especially well with PSR staining (r^2^ = 0.819; *p* < 0.0001, Figure 2f), suggesting it may be the most accurate indicator of fibrosis.

### 2.3. [^68^Ga]FAPI and [^18^F]FDG PET Reflects Different Pathological Changes of Pulmonary Fibrosis

**Figure 3:**
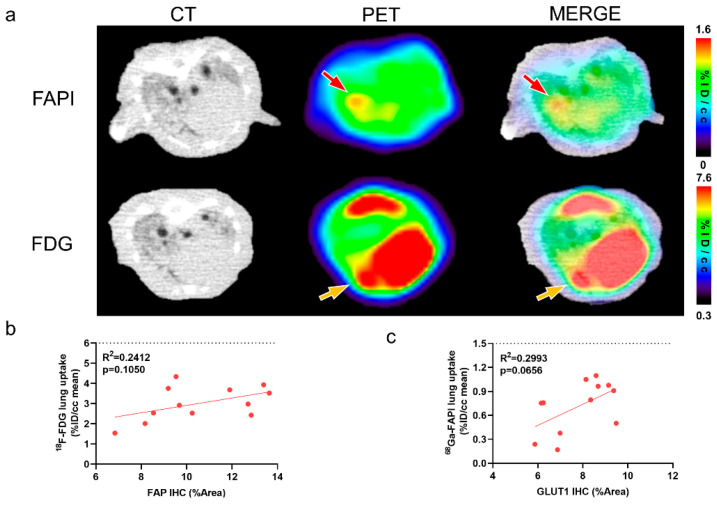
CT, [^18^F]FDG and [^68^Ga]FAPI reflect different pathological changes of pulmonary fibrosis. (**a**) The same consolidation foci displayed on the CT in same mice at 21 days after modelling demonstrated different location and degree uptake on [^68^Ga]FAPI and [^18^F]FDG PET images. Red arrows point to the high [^68^Ga]FAPI uptake lesion; whereas, yellow arrows mark the relatively high [^18^F]FDG uptake lesion. (**b**) Correlation between [^18^F]FDG lung uptake (%ID/cc mean) and percentage positive area (%Area) of FAP immunohistochemistry (IHC) in BLM groups. (**c**) Correlation between [^68^Ga]FAPI lung uptake (%ID/cc mean) and percentage positive area (%Area) of GLUT1 in BLM groups. *p* < 0.05 represents statistically significant.

Figure 3a shows a representative image of IPF consolidation lesions that exhibited different location and degrees on [^18^F]FDG and [^68^Ga]FAPI PET images. FAP expression did not significantly correlate with [^18^F]FDG lung uptake (Figure 3b). Similarly, GLUT1 expression did not correlate with [^68^Ga]FAPI uptake (Figure 3c). These results further confirm that different images reflect different changes of diseases at the molecular level.

### 2.4. CT Imaging Confirms Effective Pirfenidone Treatment

**Figure 4:**
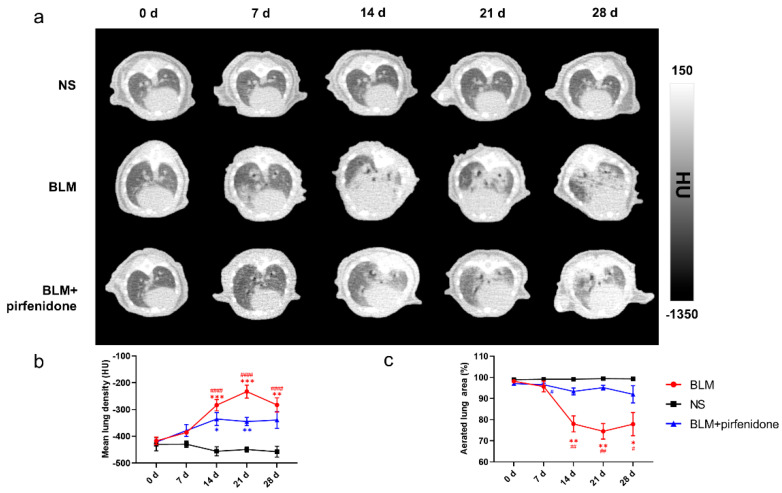
Serial lung CT images in different modelling groups. (**a**) Representative serial lung CT images of mice on day 0, 7, 14, 21, and 28 in the control group, BLM group, and treatment group. (**b**) Mean lung density quantified on CT images at different time points. (**c**) The percentage of aerated lung area in the control group (shown as NS), BLM, and BLM+ pirfenidone groups. (#) represents the statistical comparison with day 0 for each group; * (#) *p* < 0.05, ** (##) *p* < 0.01, *** *p* < 0.001, and #### *p* < 0.0001.

Figure 4 shows the serial lung CT images in different groups. In the BLM group, lung consolidation was visualized on day 7, peaked on day 21, and then decreased. In contrast, lung consolidation was less and remained relatively stable from day 14 to 28 in the BLM + pirfenidone group (Figure 4a). MLD was highest on day 21 in the BLM group (−232.833 ± 24.190 HU); in the BLM + pirfenidone group, it was highest on day 14 (−335.6 ± 23.961 HU; Figure 4b). Aerated lung area percentage over time demonstrated the same trend as seen in the CT images (Figure 4c). These findings indicate that pirfenidone reduces fibrosis progression.

### 2.5. [^18^F]FDG PET Reflects Pirfenidone Treatment

**Figure 5:**
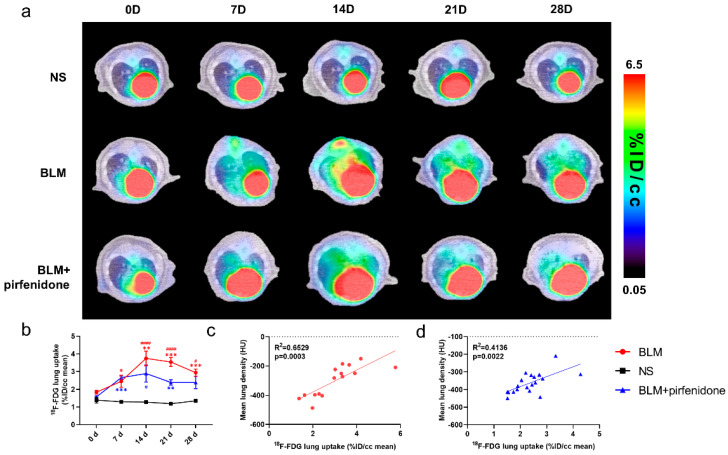
Serial lung [^18^F]FDG-PET/CT images in different groups. (**a**) Representative serial lung [^18^F]FDG-PET/CT images on day 0, 7, 14, 21, and 28 in the control group (NS), BLM group, and BLM + pirfenidone treatment group. (**b**) The lung uptake of [^18^F]FDG at different time points in different groups. The correlation between mean lung density (MLD) and [^18^F]FDG lung uptake in the BLM (**c**) and BLM + pirfenidone group (**d**). (*) represents the statistical comparison with the control group, and (#) represents the statistical comparison with day 0. * (#) *p* < 0.05; ** *p* < 0.01; *** *p* < 0.001; #### *p* < 0.0001.

Serial lung [^18^F]FDG-PET/CT images are shown in Figure 5. The peak of [^18^F]FDG lung uptake was observed in the BLM (3.745 ± 0.413 %ID/cc) and BLM + pirfenidone (2.887 ± 0.477 %ID/cc) groups on day 14 (Figure 5a,b). [^18^F]FDG lung uptake significantly correlated with MLD in both groups (Figure 5c,d). The lower degree of [^18^F]FDG uptake in the BLM + pirfenidone group and the excellent correlation between uptake and MLD demonstrates that [^18^F]FDG PET can reflect the response to pirfenidone treatment.

### 2.6. [^68^Ga]FAPI PET Accurately Reflects Efficacy of Pirfenidone

**Figure 6:**
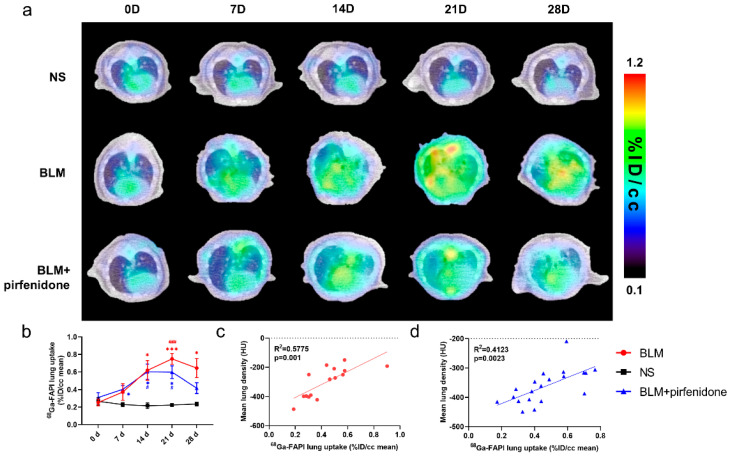
[^68^Ga]FAPI PET enables accurate evaluation of lung anti-fibrotic therapies with pirfenidone. (**a**) Representative serial lung [^68^Ga]FAPI PET/CT images of mice on day 0, 7, 14, 21, and 28 in the control group, BLM group, and BLM + pirfenidone treatment group. (**b**) The lung uptake of [^68^Ga]FAPI at different time points. The correlation between mean lung density (MLD) and [^68^Ga]FAPI lung uptake of mice in the BLM group (**c**) and BLM + pirfenidone group (**d**). (*) represents the statistical comparison with the control group at each time point; (#) represents the statistical comparison with day 0 for each group. * (#) *p* < 0.05; *** (###) *p* < 0.001.

Figure 6 shows the [^68^Ga]FAPI PET/CT images in different groups at different time points. [^68^Ga]FAPI lung uptake in the BLM group showed the same trend as lung consolidation on CT. Uptake peaked on day 21 (0.749 ± 0.062 %ID/cc; Figure 6a). In the BLM + pirfenidone group, peak [^68^Ga]FAPI lung uptake was lower and occurred on day 14 (0.602 ± 0.088 %ID/cc). Moreover, uptake declined and then remained significantly lower on days 21 and 28 (Figure 6b). [^68^Ga]FAPI lung uptake significantly correlated with MLD in both groups (Figure 6c,d). *p*-values for statistical comparisons of tracer uptake values at different times have been included in the Supplemental Materials Information (Supplemental Materials Table S2).

**Figure 7:**
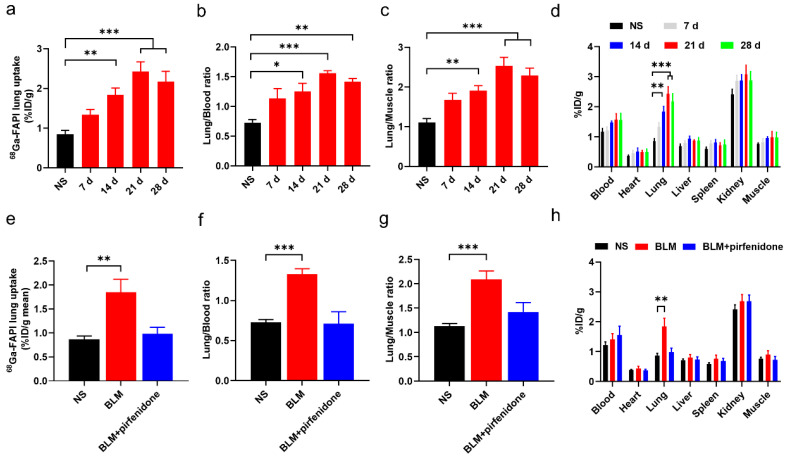
Biodistribution of [^68^Ga]FAPI in mice of different groups. (**a**–**c**) Lung uptake of [^68^Ga]FAPI (**a**), the uptake ratio of lung-to-blood (**b**) and the uptake ratio of lung-to-muscle (**c**) at day 0, 7, 14, 21, and 28 in the control group (NS) group and BLM group. (**d**) Biodistribution of [^68^Ga]FAPI of different organs of mice in NS group and BLM group. (**e**–**g**) [^68^Ga]FAPI lung uptake (**e**), the uptake ratio of lung-to-blood (**f**) and the uptake ratio of lung-to-muscle (**g**) at day 28 in the control group (NS), BLM group, and BLM + pirfenidone group. (**h**) Biodistribution of [^68^Ga]FAPI at day 28 in different groups. The value of the control group was obtained at day 28. Results are presented as mean ± SEM. * *p* < 0.05; ** *p* < 0.01; *** *p* < 0.001.

The biodistribution study results agreed with these findings (Figure 7). [^68^Ga]FAPI lung uptake, lung-to-blood ratio, and lung-to-muscle ratio were significantly higher in the BLM group than the control group (Figure 7a–d). However, they did not significantly differ between the BLM + pirfenidone and control groups (Figure 7e–h). These findings suggest that [^68^Ga]FAPI is a feasible tracer for lung fibrosis diagnosis, and the response to pirfenidone can be monitored using [^68^Ga]FAPI PET.

### 2.7. Pathological Findings Confirm Imaging Results and Validate Pirfenidone Therapy

**Figure 8:**
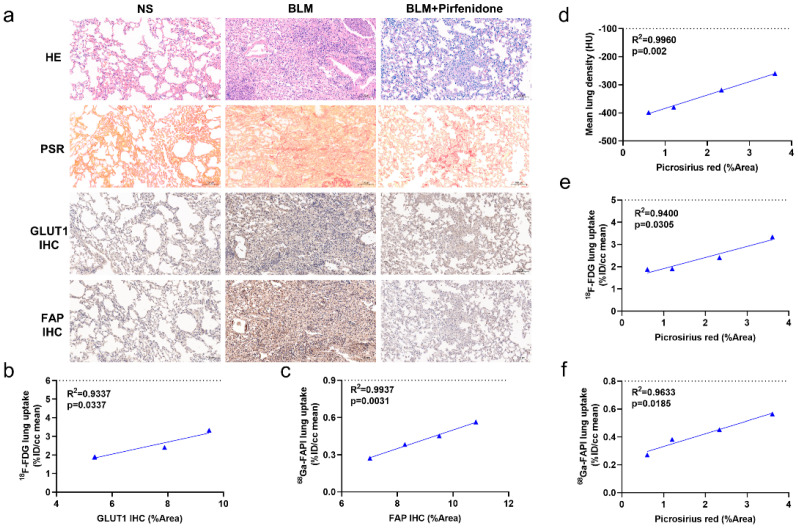
Pathological findings and the relationships with CT, [^18^F]FDG, and [^68^Ga]FAPI PET images. (**a**) Lung hematoxylin-eosin (HE) staining, picrosirius red staining, immunohistochemical staining of glucose transporters 1 (GLUT1), and fibroblast-activation protein (FAP) in the control group (NS), BLM group, and BLM + pirfenidone group. The pathological sections of the control group were obtained at day 28. (**b**) Correlation between [^18^F]FDG lung uptake (%ID/cc) and the percentage positive area (% Area) of GLUT1 immunohistochemistry staining of the mice in the treatment group. (**c**) Correlation between [^68^Ga]FAPI lung uptake (%ID/cc) and percentage positive area (% Area) of FAP immunohistochemistry staining of the mice in the treatment group. (**d**–**f**) Correlation between mean lung density, lung [^18^F]FDG and [^68^Ga]FAPI uptake (%ID/cc), and picrosirius red staining (%Area) of the mice in the treatment group.

Figure 8a shows substantial epithelial hyperplasia and strong collagen staining in tissue sections of BLM group mice, confirming successful establishment of the IPF model. In contrast, epithelial hyperplasia and collagen staining were mild in the BLM + pirfenidone group. Interestingly, increased GLUT1 staining was observed in inflamed areas of diseased lungs, and increased FAP staining was seen in fibrotic foci (Figure 8a). Quantitative values for PSR staining and GLUT1 and FAP expression were significantly higher in the BLM group than the BLM + pirfenidone and control groups (Figure 8d–f). However, no significant differences were observed between the control and BLM + pirfenidone groups (Supplemental Materials Figure S1d–f). Strong correlations were found between imaging findings and quantitative values in the BLM + pirfenidone group, suggesting that CT and [^18^F]FDG and [^68^Ga]FAPI PET can reflect corresponding pathological changes of lung fibrosis during its onset, progression, and remission (Figure 8b–f). [^68^Ga]FAPI PET appears particularly able to assess FAP expression level and collagen content and monitor treatment response to pirfenidone.

## 3. Discussion

Idiopathic pulmonary fibrosis (IPF) is a silent but progressive disease which is not easy to diagnose and monitor. In this study, we prepared IPF animal models, and treated with commonly used anti-fibrotic drugs. Three imaging modalities were continuously performed to monitor the lung changes during the development and treatment of IPF. The results of imaging and pathological findings showed that CT, [^18^F]FDG PET, and [^68^Ga]FAPI PET could be used for diagnosis and treatment evaluation of IPF from anatomical changes, glucose metabolism, and fibrosis, respectively. Because of selectively targeting FAP in remodeling tissues in IPF, [^68^Ga]FAPI PET could be used to diagnose IPF in the fibrosis stage and effectively assess treatment response.

CT imaging is widely used to diagnose pulmonary fibrosis and monitor its treatment. [^18^F]FDG PET may also have a role. Although [^18^F]FDG PET has prognostic value in fibrotic interstitial lung diseases [33,34,35], it only reflects metabolic status, not the response to treatment [35]. [^68^Ga]FAPI PET/CT is also a promising imaging modality [36]; however, its value in treatment monitoring has not been well-studied. Uptake of [^68^Ga]FAPI is associated with disease progression in patients with systemic sclerosis-associated interstitial lung disease. Furthermore, treatment of these patients with the antifibrotic drug nintedanib results in decreased [^68^Ga]FAPI uptake [37]. [^68^Ga]FAPI appears to be superior to [^18^F]FDG for therapeutic monitoring.

In our BLM-induced pulmonary fibrosis mouse model, CT was able to monitor disease severity and progression. As in previous studies, CT imaging was in good agreement with histological findings [38,39]. The highest MLD and lowest aerated lung area appeared on day 21, when histological examination showed inflammatory cell infiltration and alveolar collapse were greatest. These increases and decreases in MLD and aerated lung area reflected lung consolidation changes, which were caused by the progression of pulmonary inflammation and fibrosis from mild to severe. Of note, the high correlation between MLD and PSR staining also demonstrated that MLD and aerated lung areas can quantify disease progression over time.

Several previous studies have reported that [^18^F]FDG PET has a role in pulmonary fibrosis diagnosis and prognostication [8,33,35]. In our study, [^18^F]FDG uptake and GLUT1 expression were strongly correlated. [^18^F]FDG lung uptake initially increased and then decreased, which has been previously reported [40]. A large number of inflammatory cells with a high expression level of GLUT1 were observed in the lungs of BLM group mice. Then, between day 21 and 28, fibroblasts with relatively lower GLUT1 expression became the major component. This finding is consistent with previous studies that reported inflammatory cells and myofibroblasts expressed GLUT1 in pulmonary fibrosis [41,42]. [^18^F]FDG PET appears to demonstrate changes in GLUT1 expression levels that reflect inflammatory cell infiltration. Therefore, it may be useful to detect pulmonary fibrosis in the inflammatory stages.

Our PSR staining results identified the course of collagen deposition over time in the BLM-induced pulmonary fibrosis model. Many previous studies have shown that activated fibroblasts play a pivotal role in the production and deposition of interstitial collagen and other extracellular matrix materials [43]. FAP is a specific surface biomarker of active fibroblasts in fibrotic tissue [30,36]. In our study, the number of fibroblasts with high expression levels of FAP were highest on day 21. Furthermore, FAP expression and [^68^Ga]FAPI lung uptake were positively correlated, indicating that [^68^Ga]FAPI can reflect the number and distribution of fibroblasts expressing FAP. Peak uptake occurred when the largest number of activated fibroblasts appeared, suggesting that [^68^Ga]FAPI can visualize fibroblasts in mid- and late-stage pulmonary fibrosis.

Lung consolidation areas showed varying degrees of [^68^Ga]FAPI and [^18^F]FDG uptake. Areas with high [^18^F]FDG uptake might have been in an inflammatory phase dominated by inflammation cells with a high level of GLUT1 expression. In contrast, areas with high [^68^Ga]FAPI uptake might have been in a fibrosis phase dominated by activated fibroblasts expressing FAP. However, CT is sensitive to reflect morphological changes in which it is difficult to accurately distinguish two pathological changes at the cellular level. GLUT1 is expressed in inflammatory cells and fibroblasts, both of which are associated with collagen deposition. This may explain the correlation between [^18^F]FDG uptake and PSR staining and why [^18^F]FDG PET did not accurately reflect fibroblast changes. Because [^68^Ga]FAPI and [^18^F]FDG have different targets, it is not difficult to understand the lack of correlation between [^68^Ga]FAPI lung uptake and GLUT1 expression and between [^18^F]FDG lung uptake and FAP expression. The two imaging tracers appear to specifically detect different pathological changes.

Pirfenidone ameliorates lung fibrosis via its actions on inflammatory cells and fibroblasts and inhibition of inflammatory cytokines and growth factors [44,45]. Our study demonstrated that CT and [^18^F]FDG and [^68^Ga]FAPI PET could show changes in pulmonary fibrosis after pirfenidone treatment. Suppression of inflammatory cells and fibroblasts resulted in lower collagen deposition, as shown by PSR staining. As shown in studies comparing [^18^F]FDG and [^68^Ga]FAPI PET/CT before and after immunosuppressive treatment in patients with IgG4-related disease [28], [^68^Ga]FAPI PET/CT can demonstrate changes in fibrotic activity that are not detected by [^18^F]FDG. Therefore, [^68^Ga]FAPI PET/CT can provide data relevant to treatment decision-making, particularly for patients in the middle and advanced stages of pulmonary fibrosis.

This study has several limitations. The BLM-induced pulmonary fibrosis mouse model does not accurately reflect chronic lung fibrosis in humans [46,47], and the pathological changes differ between mice and humans. Therefore, human studies are needed to confirm our findings. In addition, our sample size was small. Another limitation is insufficient histopathological investigation on the inflammatory cells and fibroblasts, the further multiplex immunofluorescence staining of biomarkers of inflammatory cells and fibroblasts will show more unequivocal mapping of immunocytes and fibroblasts and then help us realize a more accurate location and identification of FAP-positive and GLUT1-positive cells. Meanwhile, the further combination of autoradiography and immunostaining studies will effectively reveal the relationship between intrapulmonary distribution of tracers and pathologic changes. Furthermore, due to the finite resolution of CT in murine models, some imaging features of pulmonary fibrosis, such as honeycomb changes, were ignored, which decreases its detecting power in pulmonary fibrosis.

## 4. Materials and Methods

### 4.1. Animal Experiments

**Figure 9:**
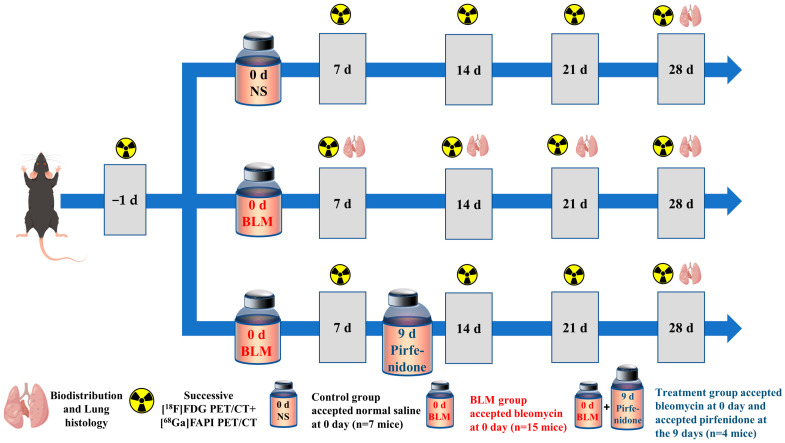
Animal grouping and experimental design. NS denotes control group; BLM denotes mice receiving bleomycin for IPF model establishment; another group of mice with IPF were treated with pirfenidone for treatment.

All animal experiments were performed in accordance with the Guidelines for the Care and Use of Laboratory Animals and approved by the Institutional Animal Care and Use Committee of the Union Hospital, Tongji Medical College, Huazhong University of Science and Technology. Eight-week-old male C57/Bl6 mice were randomly allocated into one of three experimental groups: control, bleomycin (BLM), and BLM + pirfenidone. On day (D) 0, the mice in the BLM and BLM + pirfenidone groups received a single intratracheal injection of BLM (2 mg/kg) to establish the IPF model, while the control group received saline. The BLM and BLM + pirfenidone groups collectively comprised the IPF group. Mice in the BLM + pirfenidone group also received oral pirfenidone (400 mg/kg/day) from days 9 after bleomycin instillation until the end of the experiment on day 28. Figure 9 shows the experimental schedule.

### 4.2. PET Imaging and Biodistribution Studies

CT and PET were performed using a small-animal PET/CT scanner (Novel Medical, Beijing, China). Briefly, mice were anaesthetized using 1.5% isoflurane. Static PET imaging (10 min; energy window, 250–750 keV; time window, 1.2 ns; resolution, 1.3 mm) was obtained about 50 min after intravenous injection of 6.0 MBq of [^68^Ga]FAPI or 4.0 MBq of [^18^F]FDG. Then, CT was performed (50 kV; 100 μA; ^18^0 μm resolution). All mice received baseline CT and PET scans with [^18^F]FDG and [^68^Ga]FAPI before BLM or saline administration. After model initiation at day 0 (D0), the mice underwent longitudinal [^18^F]FDG and [^68^Ga] PET/CT at D6/D7, D13/D14, D20/D21, and D27/D28. In the BLM group, some of the mice were sacrificed after [^68^Ga] PET/CT scan, *n* = 4 at D7/D14/D28, *n* = 3 at D21. And in the control and treatment group, 4 mice were sacrificed at D28. Their blood, lung, heart, liver, spleen, kidney, and muscle were harvested for ex vivo measurement of radioactivity using a γ-counter (2470 Automatic Gamma Counter WIZARD; PerkinElmer, Norwalk, CT, USA). Radioactivity is expressed as percentage of injected dose per cubic centimeter of tissue (%ID/cc).

### 4.3. Imaging Analysis

Imaging analysis was performed using Carimas 2.10 software (Turku PET Centre, Turku, Finland) to draw three-dimensional regions of interest (ROIs) in CT and PET images. To quantify the radioactivity of [^18^F]FDG and [^68^Ga]FAPI in aerated and non-aerated lung areas, normal lung density areas (less than −100 Hounsfield units [HU]) and high lung density areas (−100 to 300 HU) were drawn using the software’s semi-automatic segmentation function.

### 4.4. Hematoxylin-Eosin (HE) Staining, Immunohistochemistry, and Quantification

The lungs were fixed in 4% paraformaldehyde, dehydrated using gradient alcohol, embedded in paraffin, and cut into 4-μm slices for HE, picrosirius red (PSR), and FAP and GLUT1 immunohistochemistry (IHC) staining. Staining results were observed under a slide scanner (Pannoramic DESK, P-MIDI, P250; 3D HISTECH, Budapest, Hungary). Image J 1.8.0 software was used to quantify the percentage of area that stained positive. Detailed steps are provided in the Supplemental Materials.

### 4.5. Statistical Analysis

Statistical analyses were performed using Prism 8.0 software (GraphPad Software, San Diego, CA, USA). Data were compared using parametric analysis of variance. *p* < 0.05 was considered significant.

## 5. Conclusions

CT, [^18^F]FDG PET, and [^68^Ga]FAPI PET can monitor disease progression and treatment response in a preclinical mouse model of pulmonary fibrosis. [^68^Ga]FAPI PET/CT reflects expression of FAP in fibrotic lungs, which enables precise assessment of fibrosis and response to treatment.

## Data Availability

The raw data supporting the conclusions of this article will be made available by the authors on request.

## Supplementary Materials

The supporting information can be downloaded at: https://www.mdpi.com/article/10.3390/ph17060726/s1. Figure S1. The quantitative values of pathological sections. Table S1. The *p* values of the multiple comparisons of the max values of MLD, [18F]FDG and [68Ga]FAPI lung uptake, the positive area (%) of picrosirius red staining, GLUT1 IHC staining and FAP IHC staining of mice in BLM group with values in NS group and values at other timepoints in BLM group. Table S2. The *p* values of the multiple comparisons of the max values of MLD, [18F]FDG and [68Ga]FAPI lung uptake of mice in BLM group and BLM + pirfenidone group with values at other timepoints.
