# Supplementary material for: [68Ga]FAPI PET for Imaging and Treatment Monitoring in a Preclinical Model of Pulmonary Fibrosis: Comparison to [18F]FDG PET and CT
Source: Pharmaceuticals (Basel). 2024 Jun 3;17(6):726. doi: 10.3390/ph17060726 (PMC11206307; doi:10.3390/ph17060726)
Supplement: Supplementary file 1 [file pharmaceuticals-17-00726-s001.zip › pharmaceuticals-2988452-supplementary.pdf]

Supplementary Information

Hao Ji [1,2], Xiangming Song [1,2], Xiaoying Lv [1,2], Fuqiang Shao [1,2], Long Yu [1,2], Yangmeihui Song [1,2], Wenyu Song [1,2], Pengxin Qiao [1,2], Yongkang Gai [1,2,3], Dawei Jiang [1,2,3*], Xiaoli Lan [1,2,3*]

1 Department of Nuclear Medicine, Union Hospital, Tongji Medical College, Huazhong University of Science and Technology, Wuhan, Hubei, 430022, China

2 Hubei Key Laboratory of Molecular Imaging, Wuhan, Hubei, 430022, China.

3 Key Laboratory of Biological Targeted Therapy of the Ministry of Education, Wuhan, 430022, China.

***Corresponding authors:**

Xiaoli Lan, MD, Ph.D

Address: Jiefang Ave 1277, Wuhan, Hubei Province 430022, China.

TEL: +86-27-83692633

E-Mail: xiaoli_lan@hust.edu.cn

Dawei Jiang, PhD

Address: Jiefang Ave 1277, Wuhan, Hubei Province 430022, China.

TEL: +86-27-83692633

E-Mail: (daweijiang@hust.edu.cn)

Conflict of interest:

All authors have no conflicts of interest regarding this manuscript.

Hematoxylin-Eosin staining, PSR staining, Immunohistochemistry staining and quantification.

H&E Staining. When biodistribution was accomplished, lung tissues were fixed in 4% paraformaldehyde. After being encased in paraffin and cut into 5 mm slices, the sections were stained with H&E staining solution according to the standard H&E protocol.

PSR staining. After dewaxed and rehydrated, the slices were treated in PSR staining solution (Aspen, AS1067, China) for 1.5 hours followed by hematoxylin for 2 min. Then the slices were washed in acidified water, dehydrated in graded ethanol series and cleared in xylene.

Immunohistochemistry staining. The slices were incubated with bovine serum albumin (BSA) and then incubated with rabbit anti-mouse FAP polyclonal antibody (diluted 1:200, Thermo Fisher Scientific, PA5-99313, USA) and rabbit anti-mouse GLUT1 polyclonal antibody (diluted 1:200, Proteintech, 21829-1-AP, China) overnight. HRP-labeled goat anti-rabbit IgG (diluted 1:200, Aspen, AS-1107, China) was added as a secondary antibody and incubated for 30 min. Finally, tissue slices were stained with 3,3′-diaminobenzidine (DAB) and hematoxylin.

Image Processing and Fiber Quantification. The photo of multiple views in tissue slices were acquired by digital slice scanner (3D HISTECH, Hungary). The percentage of positive area (% Area) for PSR and IHC staining slices were quantified using ImageJ 1.51j8 software (Wayne Rasband, National Institutes of Health, USA).

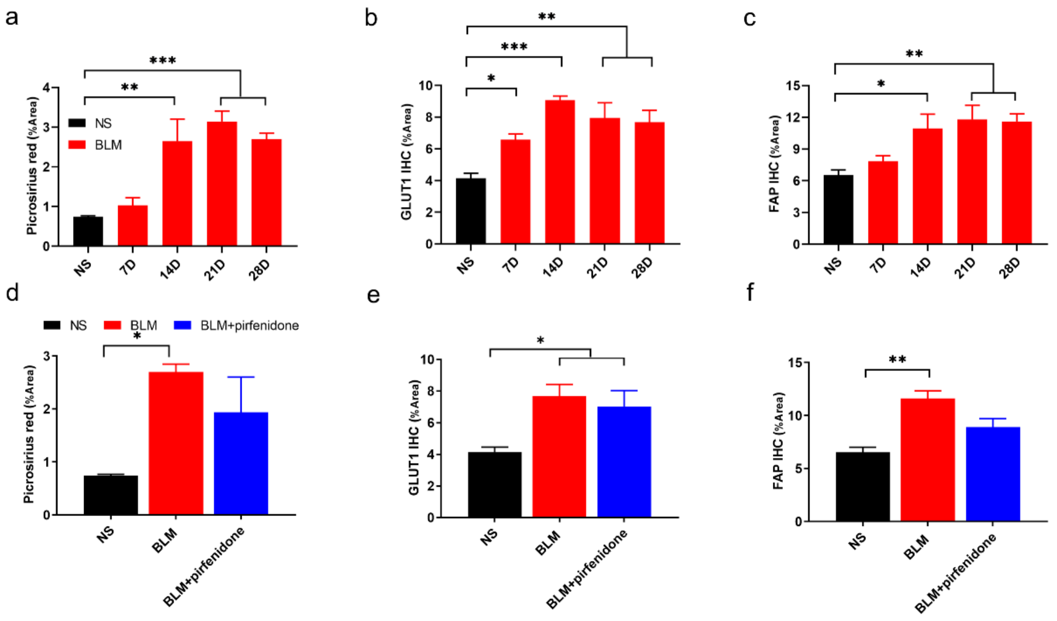

Figure S1. The quantitative values of pathological sections.

The positive area (%) of picrosirius red staining **(a)**, GLUT1 immunohistochemistry (IHC) staining **(b)** and FAP IHC staining **(c)** of mice in control group (NS) and BLMS group at various timepoints. The positive area (%) of picrosirius red staining **(d)**, GLUT1 IHC staining **(e)** and FAP IHC staining **(f)** of mice in control, BLM, and pirfenidone treatment groups at 28 days after modeling. Stars (*) are representative of statistical comparison between time points for each group. *p<0.05; **p<0.01; ***p<0.001.

	**MLD Day21**	**[18F]FDG Day 14**	**[68Ga]FAPI Day 21**	**picrosirius red Day 21**	**GLUT1 IHC Day 14**	**FAPI IHC Day 21**
**NS**	**<0.001**	**0.003**	**0.021**	**<0.001**	**<0.001**	**0.005**
**Day 0**	**0.004**	**0.038**	**0.032**	**-**	**-**	**-**
**Day 7**	**0.021**	**0.345**	**0.193**	**0.001**	**0.035**	**0.038**
**Day 14**	**0.584**	**-**	**0.997**	**0.560**		**0.909**
**Day 21**	**-**	**>0.999**	**-**	**-**	**0.484**	**-**
**Day 28**	**0.419**	**0.592**	**0.997**	**0.640**	**0.313**	**>0.999**

Supplementary Table S1. The *P* values of the multiple comparisons of the max values of MLD, [^{18}F]FDG and [^{68}Ga]FAPI lung uptake, the positive area (%) of picrosirius red staining, GLUT1 IHC staining and FAP IHC staining of mice in BLM group with values in NS group and values at other timepoints in BLM group

	BLM			BLM + pirfenidone		
	**MLD Day21**	**[18F]FDG Day 14**	**[68Ga]FAPI Day 21**	**MLD Day21**	**[18F]FDG Day 14**	**[68Ga]FAPI Day 21**
**Day 0**	**<0.001**	**<0.001**	**<0.001**	**0.069**	**0.015**	**0.105**
**Day 7**	**<0.001**	**0.001**	**0.002**	**0.685**	**0.955**	**0.389**
**Day 14**	**0.153**	**-**	**0.513**	**0.996**	**-**	**>0.999**
**Day 21**	**-**	**0.939**	**-**	**-**	**0.613**	**-**
**Day 28**	**0.164**	**0.061**	**0.691**	**0.999**	**0.610**	**0.457**

Supplementary Table S2. The *P* values of the multiple comparisons of the max values of MLD, [18F]FDG and [68Ga]FAPI lung uptake of mice in BLM group and BLM + pirfenidone group with values at other timepoints.
